# Technological advances in preclinical meta-research

**DOI:** 10.1136/bmjos-2020-100131

**Published:** 2021-07-25

**Authors:** Alexandra Bannach-Brown, Kaitlyn Hair, Zsanett Bahor, Nadia Soliman, Malcolm Macleod, Jing Liao

**Affiliations:** 1 Berlin Institute of Health, QUEST Center, Charité Universitätsmedizin Berlin, Berlin, Germany; 2 Institute for Evidence-Based Practice, Bond University, Robina, Queensland, Australia; 3 Centre for Clinical Brain Sciences, The University of Edinburgh Edinburgh Medical School, Edinburgh, Scotland, UK; 4 Pain Research; Faculty of Medicine, Department of Surgery and Cancer, Imperial College London, London, Greater London, UK

**Keywords:** systematic review, automation techniques, meta-research, preclinical meta-research

## Introduction

Metaresearch is a scientific field involving the study of research itself. It has been applied to clinical trials since the 1980s,^1^ but has only become an emerging discipline over the last decade in the preclinical field. The primary tool of metaresearch is the systematic review, which uses predefined methods to provide a transparent and comprehensive summary of the evidence relating to a research question. A systematic review is defined as ‘a review that uses explicit, systematic methods to collate and synthesize findings of studies that address a clearly formulated question’.^2^ Systematic reviews allow for evaluation of methods and comprehensiveness of reporting, to assay likelihood of reproducibility and potential for translatability to subsequent domains of research and can investigate the impact of incentives on primary research. This, in turn, allows for a more rigorous understanding of what makes research reliable, and how research can be improved,^3^ while driving evidence-based decisions for future research.^4 5^

Systematic reviews typically comprise several steps. Before beginning a systematic review, it is recommended that the author team develop a protocol, which defines the research question and the methods that will be used to conduct, analyse and report the findings of the review. The research question determines the resources required to complete the review, the broader the question and the larger the field the more resource intensive a review will be. The search strategy is developed to identify as much potentially relevant literature as possible, often involving searching of multiple databases. Following database searches, deduplication (if searching multiple database with overlapping coverage), the unique search results are screened for inclusion or exclusion. Full-text retrieval may be conducted before or after title and abstract citation screening. Metadata including information regarding reported study quality and design, and outcome data are then extracted from the included studies. A qualitative summary may be used to synthesise reported risk of bias and study design information^6^ and, where appropriate, a meta-analysis can be performed.

Systematic review methodology was largely developed for clinical evidence synthesis and has more recently been adapted to assess preclinical evidence.^7^ In this context, we use ‘preclinical’ to refer to primary experiments conducted in animals which model physiological mechanisms relevant to human health and/or test treatments to improve human health (please refer to the Glossary (table 1) for commonly used terms throughout the article). However, in comparison to clinical systematic reviews, preclinical systematic reviews present their own challenges. There is usually a higher volume of studies to summarise. For example, in preclinical neuropathic pain research, the number of articles retrieved by a systematic search rose from 6506 in 2012 to 12 614 in 2015^8^ whereas comparatively, only 129 neuropathic pain clinical trials were identified.^9^ There is also large variation in the narration used and importance given to reporting key concepts like measures taken to reduce risks of bias and Population, Intervention, Comparator, and Outcome (PICO) elements, and ultimately we are interested in abstracting a more heterogenous breadth of data to address a range of research questions. For example, outcomes of interest may range from protein levels, drug concentration, to behavioural outcomes, with each experiment reporting key experimental characteristics in different ways.

**Table 1:** Glossary of terms and abbreviations used throughout this article

Automation tool	‘Automation tool’ refers to a software application with a user interface that fully or partially automates a task conducted by systematic reviewers.^15^’
Camarades	Collaborative Approach to Meta-Analysis and Review of Animal Data from Experimental Studies. CAMARADES is an international collaboration of researchers working in preclinical systematic reviews and meta-research. Based at University of Edinburgh, it has five additional coordinating centres; BIH QUEST Center for Transforming Biomedical Research, Charité—Universitätsmedizin Berlin, Germany; University of Tasmania, Australia; SYRCLE, Radboud University Nijmegen Medical Centre, Netherlands; University of California San Francisco, United States; Ottawa Hospital Research Institute, Canada.
Deduplication	Deduplication refers to the task of removing duplicate citations from systematic searches across multiple bibliographic databases. This step is done prior to citation screening.
Full-text retrieval	Full-text retrieval refers to the task of identifying and downloading full-text publications of articles potentially relevant to the research question of a systematic review. The retrieved format may be PDF, or machine-readable such as XML or HTML.
Metadata	Metadata here refers to the structured set of elements which describe the bibliographic record (journal article, experimental report, preprint). For example, Title, Authors, Year of Publication, Journal, Issues & Volume, Abstract, Digital Object Identifier (DOI), PubMed ID or other database identifier.
Preclinical	The term ‘preclinical’ in this context refers to primary experiments conducted in animals to test treatments for human health or to model mechanisms for human health
Systematic review	A systematic review is a review that uses explicit, systematic methods to collate and synthesize findings of studies that address a clearly formulated question^2^
SyRF	CAMARADES-NC3Rs Systematic Review Facility; a free-to-use web-based software/platform to support the conduct of preclinical animal systematic reviews. Available at http://syrf.org.uk^21^

**Figure 1:**
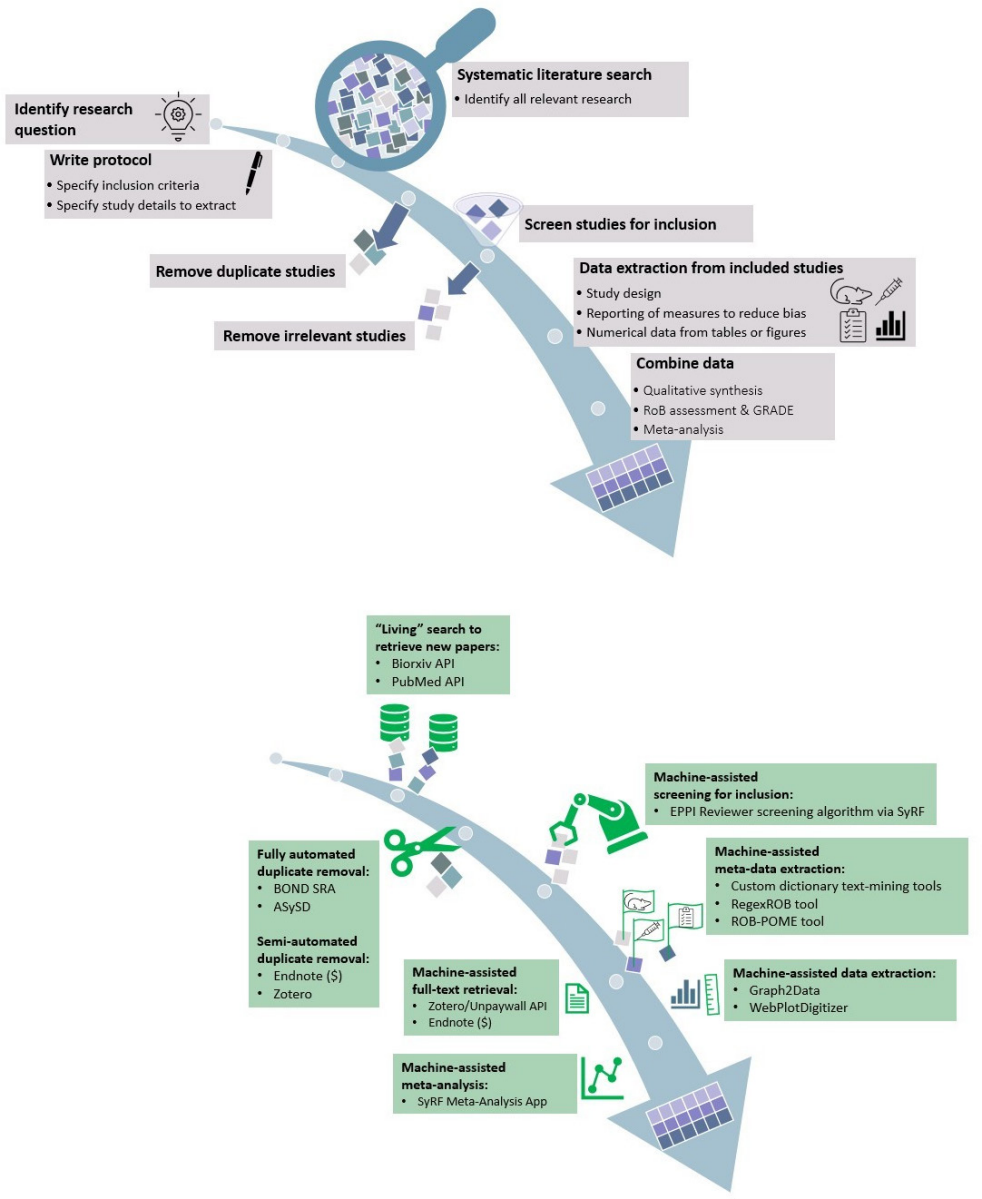
Visual representation of the steps of a systematic review in preclinical metaresearch. API, Application programming interface; EPPI, Evidence for Policy and Practice Information and coordination Centre, University College London; ROB, risk of bias; ROB-POME, risk of bias-POMEgranate; SyRF, systematic review facility.

Depending on the size of the systematic review, there are several rate and feasibility limiting steps, for example, full-text retrieval, screening for inclusion and extraction of metadata and outcome data. Database searches are completed through different online search engines, which must be repeated for every update of the systematic review. Deduplication is usually completed in a separate software, which complicates the process as well as introduces the possibility of data mishandling. Full-text retrieval involves manual searching with the help of reference management software such as Endnote (RRID:SCR_014001) or Zotero (RRID:SCR_014001); however, access is limited by pay walls and institutional subscriptions. Independent, manual dual screening and data extraction requires a significant amount of reviewer time. Numerical outcome data extraction from graphs and/or tables is one of the most time-consuming tasks that would benefit from efficient minimisation of human error and systems managing large volumes of collated data. Analysis can be complex and varies depending on the project and the extracted data. Tools and training materials to assist systematic reviewers exist but remain limited in helping inexperienced researchers to correctly choose and perform analyses. For an overview of the steps of a systematic review and where automation tools can be used, see figure 1.

Preclinical systematic reviews are vital; they serve many purposes by assessing the range and quality of the evidence. Through these reviews knowledge gaps can be identified, methodological quality improved, unnecessary duplication of experiments can be avoided, and clinical trial design can be informed.^10 11^ They increase the value of research and reduce research waste.^12^ Despite their utility, these reviews are time and resource intensive and as a result rarely up to date at the time of publishing as they cannot continuously incorporate new studies, limiting their longer-term applicability.^13^ Additionally, they are predominantly carried out by or require large amounts of support from systematic review experts.^14^ These barriers limit the feasibility of conducting a preclinical systematic review.^15^ To improve accessibility, feasibility and utility there has been a need to develop technological tools that can assist in the synthesis of evidence across the board. This article focusses on technological tools developed specifically for preclinical evidence summaries. This article will discuss the past and present technological development efforts in this domain, show the extent to which recent technological advances have impacted the preclinical systematic review pipeline and what challenges we still face. This topic remains of high relevance and while there have been considerable advancements, there is also potential to do a lot more. Many of these technological advances have been made possible in the context of the development of preclinical systematic review methodology (for key milestones see Sena & McCann, see this issue). Further examples of preclinical systematic review methodology and methodological guidance include McCann *et al*^16^; Soliman *et al*^17^; Sena *et al*^7^; Vesterinen *et al*.^18^ The ultimate aspiration is to be able to integrate new evidence into systematic reviews of existing evidence, so that decisions are informed by our most up-to-date understanding, in a ‘living evidence’ summary.

## Past

For over 20 years, several groups have been conducting systematic reviews of preclinical research. Since 2005, the Collaborative Approach to Meta-Analysis and Review of Animal Data from Experimental Studies (CAMARADES) group have both conducted their own reviews in preclinical models of human diseases in focussing on efficacy of candidate drugs, and provided support for other researchers wishing to use this approach. The Systematic Review Centre for Laboratory animal Experimentation was founded at Radboud UMC, Nijmegen, with similar dual purpose. Others groups have also become involved in the context of environmental toxicology, including the Navigation Guide from the Program for Reproductive Health and the Environment at University of California San Francisco (UCSF; since 2010), and the National Toxicology Program Office of Health Assessment and Translation (OHAT; since 2011). Of these groups, CAMARADES have been most active in the seeking technological advances to support reviews of preclinical efficacy data, and OHAT most active in the environmental toxicology field. Both have benefited from involvement in the International Consortium for the Automation of Systematic Reviews (ICASR).

Since our concern here relates to the translational value of preclinical research, the developments we describe here relate largely to work done through CAMARADES, either in developing new tools or in repurposing to preclinical systematic reviews tools developed for other systematic review domains. CAMARADES was among the first research networks to realise the potential of technology for metaresearch in the preclinical field and was born out of the vision that much like for clinical trials, there was also a need for systematic reviews of preclinical studies. Established at the Universities of Melbourne and Edinburgh, CAMARADES is an international collaboration of preclinical metaresearchers, with five national coordinating centres across the world.

**Figure 2:**
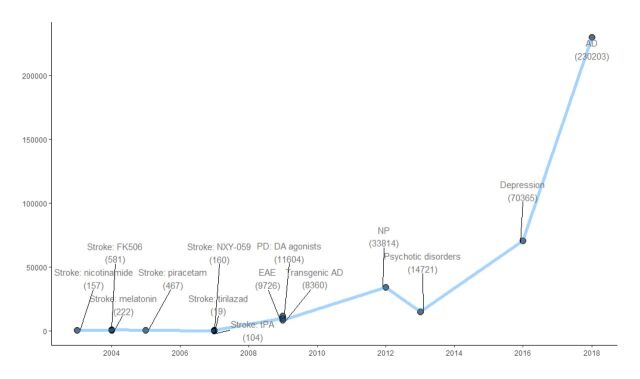
Number of unique records retrieved from preclinical systematic searches across time. Each data point represents a Collaborative Approach to Meta-Analysis and Review of Animal Data from Experimental Studies preclinical systematic review project. tPA, tissue Plasminogen Activator; EAE, experimental automimmue encephalomyelitis; PD, Parkinsons disease; DA, dopamine antaonists, AD, Alzheimer’s disease; NP, neuropathic pain.

Initially, reference management software and non-specialised tools such as Microsoft Excel were used to screen, manage and analyse studies, but the increasing size and complexity of reviews, quickly made it apparent that there was a need for more efficient project management systems. Early preclinical systematic reviews retrieved 100s of potentially relevant studies during their search of the literature^19^ (figure 2). In 2006, CAMARADES developed a preclinical systematic review database systemin Microsoft (MS) Access, which allowed reviewers to manage projects, extract structured data into fixed fields and organise these into data tables for simplified analysis. Over the years the MS Access database expanded greatly to facilitate dual citation screening, dual data extraction from full texts, and meta-analysis through custom-built queries of the data.^20^ Over 11 years, this resource supported 100s of reviewers to conduct over 50 preclinical systematic review projects. With a growing body of literature and an increasing interest in reviewing more extensive research areas, the number of unique records captured by systematic searches of the literature for various disease areas increased exponentially (figure 2). In 2013, a systematic review of animal models of multiple sclerosis identified over 9000 potentially relevant studies from the systematic search. And in 2014, a systematic review of preclinical literature on schizophrenia identified over 14 000 studies. By 2016, a project to systematically curate the evidence from animal models of depression had identified over 70 000 potentially relevant studies. The need for tools to support these large-scale systematic reviews had grown ever-more pressing.

To address concerns around system performance, scalability and accessibility of data management using the MS Access database, CAMARADES received an infrastructure award from the UK National Centre for Replacement, Refinement, and Reduction of Animals in Research (NC3Rs) to develop a web-based platform supporting preclinical systematic reviews. The Systematic Review Facility (SyRF; RRID:SCR_018907^21^) was launched on March 30th in 2016 in London. SyRF enabled researchers to efficiently manage large projects with multiple reviewers and easily collaborate with other researchers across the world. Gone were the days of collaborators using guest accounts and virtual private networks to log on to a remote MS Access database with varying degrees of success. Using SyRF, data were securely held in an encrypted cloud database and any number of collaborators across the globe could now contribute to citation screening, annotation, and data extraction simultaneously online. The application also allowed for blinded reviewing between reviewers, overcoming the previous challenge of having to create separate copies of a project for separate users. SyRF is flexible enough to support the heterogeneous nature of preclinical studies and enable researchers to extract information for a wide variety of experimental designs. SyRF continues to be a useful tool in the preclinical field and remains free at the point of use; anyone, worldwide, with an email address and internet connectivity can register and start their review straight away.

Having an online, purpose-built preclinical systematic review platform allowing for adaptable project design and management increased the ease with which reviews could be conducted. However, one major obstacle remained: performing preclinical systematic reviews still required a great deal of concentrated, manual effort to complete. The focus shifted to the development of tools which could enhance the ease and speed at which preclinical systematic reviews could be performed. Looking to other areas for inspiration, there was increasing interest in the rise in artificial intelligence and how modern computing advances may be applied to systematic review methodology. The field of preclinical systematic review started exploring how text analytics tools like machine learning and text mining may be applied to the preclinical systematic review workflow to speed up systematic review steps that require processing of textual data.

Through collaborations with computer science experts at Obuda University in Hungary, research began to investigate techniques to automate risk of bias ascertainment from articles.^22^ Further, partnerships began with the National Centre for Text-Mining in Manchester and the EPPI-Centre at University College London to investigate techniques to automate title and abstract screening.^23 24^ These research collaborations led to the development of several noteworthy tools including: the regular expression tool to automatically ascertain risk of bias from preclinical studies,^22^ machine learning algorithms for title and abstract citation screening,^23 24^ and autoretrieval of publications from online search engines (ie, ‘living searches’) integrated into the systematic review platform, SyRF (see Bahor *et al*^21^). Additionally, a key tool to support preclinical systematic reviews is the CAMARADES meta-analysis app which accepts data directly from SyRF and automates the performance of meta-analyses and visualisation of results, including quick exploration of heterogeneity between studies and publication bias, and easy creation of forest plots, funnel plots and other relevant visualisations.^25^

The automation tools developed to aid screening, assess reporting quality and calculate treatment effects in a meta-analysis, led to marked reductions in the time taken to synthesise evidence from preclinical research. Due to the development of such systems and tools, we are now able to do large-scale preclinical systematic reviews and overviews of the relevant literature. One recent example of a project that has leveraged the use of these tools to capture and process a large proportion of the biomedical literature is a systematic review of the preclinical literature on Alzheimer’s disease.^26^ A systematic search for this project in 2018 retrieved over 200 000 potentially relevant unique articles—a review of a scale that would have never been humanly possible without the help of automated tools. Projects like this demonstrate that tools are robust at this scale and lead to marked reductions in time taken to synthesise evidence from preclinical research. The question became whether there are any other areas of the systematic review workflow where similar tools may be developed to further automate tedious or error-prone manual steps of a review. Arguably some later stages of the systematic review process like data extraction can be much more time consuming and while less of a low-hanging fruit than screening of publications perhaps, machine-assisted performance of these stages of a review could make an even bigger impact on the time that it takes to complete large systematic reviews. We were working with the goal of implementing tools to have an automated end-to-end workflow for preclinical systematic review.

## Present

Since these early efforts to invite artificial intelligence to solve some of the issues related to performing systematic reviews, new tools have continued to be developed to address very specific challenges to the entirety of the systematic review pipeline. The SyRF platform continues to support preclinical systematic reviews, and provides a basis for integrating automation tools to support various stages of the review. Similar platforms, in other research domains, are also working to integrate automation tools for systematic reviews, for example, Covidence (RRID: SCR_016484) and Distiller-SR (https://www.evidencepartners.com/products/distillersr-systematic-review-software/). A comparison of systematic review software can be found in of Bahor *et al*.^3^

Current technological advancements are concentrating on answering challenges associated with the initial and final steps of the preclinical systematic review and meta-analysis pipeline to pave the way for fully automated workflows. These include focussing on the initial search of the literature—the identification and collation of a potentially suitable subset of the literature that can then be fed to machine learning algorithms developed to include studies of relevance. Automated citation retrieval via APIs or RSS feeds is now available for some bibliographic databases, including PubMed and bioRxiv, which can be accessed programmatically. As most reviews generally perform searches in multiple databases to increase the coverage of the literature and likelihood of obtaining all studies of interest, having these features available for all key electronic databases would further benefit the field. Automated deduplication tools have been developed, both as integrated tools to reference management software (Endnote, Zotero) and as separate stand-alone interfaces such as the Bond Systematic Review Accelerator deduplicator tool.^27^ More recently, the Automated Systematic Search Deduplicator^28^ has been built using gold-standard preclinical systematic search datasets; thus, this automated tool is specialised for preclinical reviews with larger search returns. Performance evaluation of fully automated duplicate removal tools across a range of datasets is ongoing.^29^

Extensive dictionaries, regular expressions and other text-mining techniques are also being developed to automate the categorisation of the articles and risk of bias ascertainment process.^30^ Custom dictionaries of key terms relevant to, for example, interventions, outcome assessments, model induction methods, in each review are being created by context experts and systematic reviews. With the help of regular expression techniques, full-text articles are automatically converted to machine-readable format and searched against the project’s custom dictionaries. The frequency of key terms across the literature, as well as co-occurrence of these terms (eg, intervention key terms and outcome assessment key terms), are used to tag, group and prioritise articles for the next steps in the systematic review process.

Some automated tools have been developed for reasons other than systematic review, but might be helpful in assessing reporting quality or risk of bias. The SciScore tool, developed in 2019 (RRID: SCR_016251), identifies reporting of rigour criteria including; Institutional Animal Care and Use Committee Statements, sex as a biological variable, antibody and organism identification, and cell line identifications and authentication.^31^ The subscription based tool uses conditional random field algorithms to detect entities.^31^ Barzooka is a tool developed by Riedel, Weissgerber and colleagues, which flags the inappropriate use of bar graphs for continuous data (RRID:SCR_018508; https://quest-barzooka.bihealth.org^32^). Being able to embed these and other tools as they are developed and validated, into systematic review pipelines will be paramount to utilising their combined benefits. The programmatic integration of tools and software developed in different programming languages and environments is guided by documentation standards and recommendations.^33^

Some of the most time-consuming tasks in preclinical metaresearch come at the end of the process when extracting numerical data from text and figures to perform a meta-analysis. Preclinical outcome data can be challenging and time consuming to gather because it is it rarely presented in a clear and easy-to-abstract format. Raw data are rarely available in a study and to extract data presented in graphs reviewers must manually measure values, which is an error prone process. Outcome data may be presented in different forms, whether that is multiple measures of the same outcome or multiple timepoints. Graph2Data, a tool developed by EPPI-Centre and CAMARADES in collaboration on the SLIM grant (MR/N015665/1),^34 35^ streamlines and semiautomates axes identification and data point identification, reducing the time required for data extraction, as well as reducing extraction and transcription errors associated with manual methods. A non-inferiority trial demonstrated that this machine-assisted approach represents a considerable time saving and is also more accurate than other human methods; for 10 participants to extract data from 23 graphs, the mean time overall was 5 min and 52 s less and 29% more accurate using the tools compared with manual methods.^34^ Ongoing work to integrate tools like this into platforms like SyRF has the potential to further facilitate the performance of preclinical meta-research.

The combination of automated search and citation retrieval from PubMed, automated deduplication, machine learning algorithms for citation screening, and the application of regular expressions and other text-mining techniques for categorisation of articles and automatic risk of bias ascertainment has made automated updating of systematic review pipelines possible. And so, the culmination of these crucial automation techniques has allowed for the application of these, and other tools, to ‘living’ evidence overviews.

The concept of a ‘living overview’ technique in preclinical metaresearch was initially applied to the literature on animal models of depression^36 37^ and has since expanded to include similar overviews on animal models of Alzheimer’s disease^26^ and more recently, COVID-19.^38^ These overviews have shown that this approach to systematic reviews can provide an up-to-date visual and interactive overview of the literature in a specific research field which has the potential to benefit many research stakeholders. For example, animal researchers or clinicians can interrogate data, and download the citations for their own use, which greatly reduces the upfront resource burden of trying to identify all relevant data in relation to a topic of interest. This is vital, because ideally, all new research should be done in the context of other research and therefore new research ventures should be based on a rigorous and systematic understanding and assessment of previous relevant literature, which is made possible by these interactive evidence summaries.

Having demonstrated feasibility that these automation techniques could be applied to preclinical metaresearch, the focus now is on routinely using these in practice. We as a field are integrating these tools, into the preclinical metaresearch workflow and into existing systematic review platforms such as SyRF, to enable widespread use. Current tools and software in our pipeline, and their corresponding systematic review step, are outlined in table 2. One of the next major challenges includes documenting and enabling these tools to be accessible to those who wish to use them as part of their reviews. To facilitate this process, the CAMARADES group rand wider automation community are developing documentation standards for tools to increase reproducibility and reporting standards, and procedures for integrating several separate tools into a single workflow.^33^ This will enable metaresearch teams across to world to combine tools based on their project needs, and to create bespoke workflows using existing platforms.

**Table 2:** Automation tools and software currently used in the systematic review of animal data workflow

Preclinical systematic review stage	Tool/software	Links to resource or reference
Protocol	SYRCLE Protocol	Publication and template^51^
Systematic search	APIs for PubMed APIs for Web of Science APIs for biorxiv and medrxiv Living Search for PubMed	PubMed API (RRID:SCR_013249) ‘wosr’ R package^52^ medrxivr R package^53^ SyRF Platform^21^
Deduplicating reference libraries	ASySD—Automated Systematic Search Deduplication	Code and web application, validation^28 29^
Citation screening (title and abstract)	SyRF platform supported by machine learning classification algorithms	21 23 24
Full-text retrieval	Unpaywall API Reference Manager Software (Endnote or Zotero) (Manual)	Unpaywall, SCR_016471 Zotero, (SCR_013784) integration with unpaywall EndNote, (SCR_014001), find full-text function ($)
Citation screening (Full text)	SyRF platform	Systematic Review Facility (SCR_018907^21^)
Metadata extraction	Custom Regular Expression Dictionaries	Regular Expression Dictionaries for document tagging and grouping^22 54^
Risk of bias	Automated detection of blinding, randomisation, and sample size calculation	Text-mining tools for automated detection^22 55^ CAMARADES Quality Checklist^19^; SYRCLE’s Risk of Bias Tool^56^
Data extraction	Machine-assisted data extraction from graphs SyRF for manual data entry, standardised forms for extracting study design characteristics, risk of bias, and quantitative outcome data	Graph2Data (34) Systematic Review Facility, (SCR_018907^21^)
Meta-analysis	SyRF Meta-Analysis App	Meta-analysis animal data in SyRF Meta-Analysis App^18 25^
GRADE, SR reporting and publication	GRADE Preclinical certainty of evidence Reporting (CAMARADES appraisal checklist, PRISMA) PRISMA Flowchart generator	GRADE Preclinical^10^ Guidelines for reporting systematic reviews and meta-analyses of animal studies.^7^ PRISMA for preclinical studies under development^57^ Systematic Review Flowchart generator^58^
Visualising findings and dissemination	Preprint of SR on Metarxiv Dashboards in Shiny R – custom created for each SR output Interactive Plots in eLIFE and F1000	Meta-Research Preprint Server^59^ R Shiny dashboards^60^ Interactive Plots with plotly in R^61^ and integration with publishing platforms, for example, F1000 Interactive Plots^62^

The symbol ‘$’ denotes a paid for software or tool.

PRISMA, Preferred Reporting Items for Systematic Reviews and Meta-Analyses.

## The increasing role of the research community in technological advances

Advances in the field can arguably only have the most impact if the whole research community is engaged in their development, their use and their incentivizement.

For one, the technological advances made to date have facilitated a novel approach of crowd sourcing to metaresearch. Online platforms, such as SyRF, support the training of emerging metaresearchers and facilitate simultaneous reviewing including screening and data extraction. The technological advances have enabled ‘crowd science’ projects such as a randomised controlled trial exploring whether implementing an ARRIVE guideline checklist in publishing improved compliance,^39^ a study on Nature’s editorial policy,^40^ and systematic review projects, with over 100 researchers from across the globe trained and actively contributing to each project.^41^ Using a crowd offers opportunities to improve the efficiency and accuracy of a systematic review as well as further develop automation technology; crowd and machine can work in mutually supportive ways. One example of this is the development of a Randomised Controlled Trial classifier, as the crowd screened more studies the classifier became more accurate, the machine removing studies that were not eligible thereby reducing the subsequent workload for the crowd.^42^ Careful consideration is required before using a crowd; a crowd requires close management and depending on the complexity of the task the crowd may require training to ensure quality. It is also useful to have means to control for quality throughout the duration of crowd involvement to allow for problems to be solved as they arise. If reliant on a crowd comprised of volunteers, the project lead should be cognisant of the impact this may have on the project timelines as well as potential harms to an individual crowd member. Notwithstanding, importantly, this crowd source approach can increase education about systematic review methodology, can reduce the divide between primary researchers and metaresearchers and is a step toward open synthesis communities.^43^ Tools and approaches such as this enable us to move towards an open synthesis community, which we envisage will lead to reduced research waste, increased collaboration, and research conducted in a timelier manner.^43^

Further approaches to increase reproducibility across the preclinical domain include the Resource Identification Initiative. Resource identifiers (RRIDs) are unique identifiers to help researchers cite the methods used in the experiment to aid reproducibility. The widespread applicability of such initiatives requires uptake by multiple stakeholders in the research community. Uptake of RRIDs by authors and journals has allowed for development of tools to identify and link reproducible methods in articles, through automated algorithms in a suite of tools called SciScore (RRID:SCR_016251). Bandrowski and team are working to expand efforts to other fields after the success with increased reporting of uniquely identifiable resources seen in antibodies and imaging software.^31^

Technology for advancing metaresearch is a growing research area, not just within preclinical medicine. Hackathons and meetings across the world are being held to build and validate tools across research domains. Notably, work from the evidence synthesis hackathons,^44^ the ICASR community,^45^ and the METAxDATA group.^46^ These hackathons enable researchers, programmers and methodologists from many different domains to openly collaborate, share ideas and approaches, work intensively on creating innovative new tools over short meetings. Hackathons are already expanding the community around automation tools and it is through these communities that shared standards and communication are increased. These communities will be key in building standards for documentation, working with funders and journals to create sustainable frameworks for maintaining tools and increasing citations and credit of automation tools used by researchers.

We are making strides towards automating sections of the preclinical systematic review process, including using crowd sourcing approaches, and the progress in automation is showcased through ‘living overview’ projects which visualise systematically aggregated data for key stakeholders. Increasing collaborative work within the research community to bring tools seamlessly into the systematic review workflow, and to embed systematic review methodology within the primary research field will be invaluable in facilitating continuous synthesis of domain data.^43^

## Future

Looking ahead, to continue making progress using automation techniques to facilitate evidence synthesis, we must tackle several ongoing challenges and exploit emerging technological advancements to eliminate resource intensive manual tasks.

One fundamental challenge lies in the sustainability of automation tools. Continued, long-term funding for the development, maintenance, and improvement of tools is urgently required to ensure they remain useful and useable by the community. However, automation tools built with grant money which then become obsolete due to code not being available, tools not being shared or maintained is wasteful. Further, it is clear that tools without a user-friendly interface or documentation to support their use present a huge barrier to researchers without technical/coding experience. In future, these challenges may be overcome by having indexed repositories of automation tools with minimum required information on the tool, external validation of the tool to ensure validity across use cases, data toolkits and demonstrations to aid users of the tools, and the ability for cross-platform integration via the use of APIs.^33^ To accelerate tool development, there is a need to establish a community responsible for these tools. As academia is inherently project based, we see migration of researchers when projects end and this often leads to the abandonment of incomplete works, or work that is poorly documented for others to understand or use. Building a strong community with agreed standards can ensure other community members can ‘pick up’ emerging or in-development tools and ensure tools and documentation are appropriately maintained. The expansion of this community will involve many key players of the research community, including researchers, data scientists, statisticians, software developers, publishers and funders who all should be conscious of contributing to the software graveyard.

It is increasingly difficult to keep up with the pace of newly published evidence, with an ever-growing body of biomedical literature.^17^ For preclinical systematic reviewers working on high-output disease areas, this may be particularly challenging. As highlighted previously,^17^ within the field of neuropathic pain, the number of potentially relevant papers identified in clinical and preclinical systematic review searches with a similar scope were 129 and 12 614, respectively.^8 9^ While this disparity may not be the case in every domain, performing and updating preclinical reviews come with unique challenges.

Comparing the life cycle of clinical trial research to the life cycle of animal trial research, from inception to completion, we can see that in clinical research, the median interval between the ethical application and the posting of results on clinicaltrials.gov is between 5 and 7 years, as demonstrated by Blümle *et al*.^47^ To estimate this in preclinical research, we can look at publications which acknowledge, for example, Medical Research Council (UK) as a funder and an intention to conduct animal research in Gateway to Research (https://gtr.ukri.org/). We identified the median interval between grant awarded and publication was 1 year (0–3 year range); we estimate approximately 12 months per experiment. If the median life cycle of animal research is 1 year compared with the median life cycle of clinical research being 6 years, the challenges regarding updating and maintaining a systematic review of the literature are very different. If a clinical systematic review is 3 years out of date, that is half the life cycle of clinical research project. If a preclinical systematic review is 3 years out of date, that is 3 life cycles of the average research project. Therefore, more than ever, automation tools and software are required to help stay up to date with the preclinical literature as the tempo varies greatly between domains.

To keep up with the life cycle of preclinical research, we will become more and more reliant on ‘living’ reviews and ‘living’ overviews, a need for several stakeholders including researchers, grant assessors and funders. With the current tools, ‘living’ reviews are still reliant on a number of manual, resource-intensive steps, such as search retrieval from bibliographic databases without programmable interfaces or with expensive pay-walled access to APIs. ‘Living’ reviews are still clunky and require a lot of work. ‘Living’ reviews in the preclinical sphere need to accumulate and synthesise data in real time. In future, we need to focus on opening these bottlenecks with software to ensure these automatic synthesises are smooth, ensure algorithms to learn from updated data as it becomes available, to allow for systematic reviews that are not just living but alive.

While we have come a long way with the tools that are already being developed, there continue to be some major challenges which we are exploring further. One of these is PDF retrieval of publications. Full-text information about a publication is key to ascertaining its relevance to a research question and to extracting information to use in systematic summaries and meta-analyses. Mostly, full-text information is presented by publishers in the form of a PDF, often with institutional subscription barriers. Currently, human reviewers using tools like Endnote and Zotero can automatically retrieve a rough average of 85% of full-text articles required for preclinical systematic reviews, depending on institutional subscription, subject area, and date of publication. Either the full-text article is available in a machine-readable format such as euPMC XML format, open access PDFs, or PDFs behind a subscription pay-wall. To retrieve full-text information for systematic reviews via reference management software is time consuming and requires a human reviewer to search for PDFs of approximately 200 citations at a time. Universal automatic PDF retrieval, with institutional ezproxy log-in, as a plug-in to platforms such as SyRF would be an invaluable addition to the systematic review pipeline, and will remove the manual step of taking data back into Endnote and searching for PDFs 200 at a time. In future, wide and reliable application of rapid novel innovations in machine learning and text-mining in biomedical science will require the full-text article and accompanying information to be made freely available, through open access, in a machine-readable format (eg, Europe PubMed Central). Without this, we will run into the same bottlenecks that we have always faced; difficulty in accessing PDFs and difficulty in converting PDFs into machine readable formats. Until we achieve this, we will not be able to maximise the use and advantage of these technological developments to our advantage in biomedical metaresearch as other areas have (finance, bioinformatics, genetics, physics).

Going forward, there are emerging tools that may change the way that we conduct preclinical metaresearch. Tools such as Microsoft academic graph, a database and network (or ‘graph’) of academic documents aiming to index all published articles, patents, and abstracts, and their related-ness,^48^ and Scite,^49^ which identifies supporting and opposing research findings, may in fact change the way systematic review methodology is applied to preclinical research questions as well as having the potential to facilitate the synthesis of evidence. If tools like Microsoft Academic Graph are freely available to all researchers, a systematic search will only need to be conducted in one database, updating the evidence in a systematic review can occur automatically based on ‘conceptual related-ness’ to included studies.^50^ Steps in the typical systematic review workflow, such as deduplication of searches, will then be replaced with other steps that ensure high quality.

As technological advances adapt and evolve, so too must our field of preclinical metaresearch, for the two to become more closely linked in future. We need to work more closely together to see technology efficiently assist the systematic overviews of our knowledge, to improve reproducibility of future research and to facilitate the translatability of preclinical findings to achieve our ultimate goal of improving human health.

## Data Availability

Data sharing not applicable as no datasets generated and/or analysed for this study.
